# Understanding individual, family and community perspectives on delaying early birth among adolescent girls: findings from a formative evaluation in rural Bangladesh

**DOI:** 10.1186/s12905-020-01044-z

**Published:** 2020-08-10

**Authors:** Ghazaleh Samandari, Bidhan Krishna Sarker, Carolyn Grant, Nafisa Lira Huq, Aloka Talukder, Sadia Nishat Mahfuz, Lily Brent, Syeda Nabin Ara Nitu, Humaira Aziz, Sara Gullo

**Affiliations:** 1 Public Health Leadership Program, University of North Carolina at Chapel Hill, Chapel Hill, USA; 2 Maternal and Child Health Division, icddr,b, Dhaka, Bangladesh; 3 CARE USA, Atlanta, USA; 4 Health System and Population Science Division, icddr,b, Dhaka, Bangladesh; 5 CARE Bangladesh, Dhaka, Bangladesh

**Keywords:** Adolescents, Girls, Youth, Delaying birth, Pregnancy, Early birth, Contraception, Bangladesh

## Abstract

**Background:**

Pregnancy among adolescent girls in Bangladesh is high, with 66% of women under the age of 18 reporting a first birth; this issue is particularly acute in the northern region of Bangladesh, an area that is especially impoverished and where girls are at heightened risk. Using formative research, CARE USA examined the underlying social, individual and structural factors influencing married girls’ early first birth and participation in alternative opportunities (such as education or economic pursuits) in Bangladesh.

**Methods:**

In July of 2017, researchers conducted in-depth interviews of community members in two sub-districts of northern Bangladesh (Kurigram Sadar and Rajarhat). Participants (*n* = 127) included adolescent girls (both married and unmarredi), husbands of adolescent girls, influential adults in the girls’ lives, community leaders, and health providers. All interviews were transcribed, coded and organized using Dedoose software.

**Results:**

Participants recognize the health benefits of delaying first birth, but stigma around infertility and contraceptive use, pressure from mothers-in-law and health provider bias interfere with a girl’s ability to delay childbearing. Girls’ social isolation, lack of mobility or autonomy, and inability to envision alternatives to early motherhood compound the issue; provider bias may also prevent access to methods. While participants agree that pursuit of education and economic opportunities are important, better futures for girls do not necessarily supersede their marital obligations of childrearing and domestic chores.

**Conclusions:**

Findings indicate the need for a multi-level approach to delaying early birth and stimulating girls’ participation in economic and educational pursuits. Interventions must mitigate barriers to reproductive health care; train adolescent girls on viable economic activities; and provide educational opportunities for girls. Effective programs should also address contextual issues by including immediate members of the girls’ families, particularly the husband and mother-in-law.

## Background

Child marriage is closely associated with early birth among adolescent girls; 90% of adolescent pregnancies in the developing world are to girls who are already married and married adolescents are more likely to experience frequent and early pregnancies than their unmarried peers [1, 2]. Adolescent girls who undergo early marriage (often defined as prior to age 18) and subsequent rapid birth are more likely to experience a host of negative physical, mental and economic outcomes including complications during pregnancy and delivery, higher rates of maternal mortality, and poor educational and economic outcomes for both themselves and their children [1–4].

A number of factors influence married adolescent girls’ ability to delay early childbearing. Entrenched social norms around gender roles, rooted strongly in community and family contexts, equate a girl’s value with her ability to procreate [4–6]. Stigma associated with rumors of infertility also yield powerful influence over young couples’ fertility choices, driving them to prove their fecundity through early birth [6, 7]. Married adolescent girls are also less likely to engage in family planning, due to a lack of knowledge of contraceptives and male-dominated partner dynamics which limit their individual ability to control the timing and frequency of pregnancy [4, 8, 9]. Furthermore, adolescents experience an inordinate number of obstacles to accessing reproductive services within the formal health system, including bias of providers, stigma around contraceptive use, and lack of physical or financial access to health facilities [10–13].

In Bangladesh, the legal minimum age for marriage is 18 for girls, but enforcement of this law is weak [8]. The current median age at first marriage is 15.8 years and 66% of Bangladeshi women report giving birth before the age of 18 [7]. Patriarchal norms and social structures make it difficult for girls to refuse sex or use contraceptives, particularly in the context of marriage [14–18]. Young wives are less able than their older peers to negotiate family planning decisions with their husbands and extended family. This lack of agency leaves young brides unable to time and space their pregnancies in a way that can improve their health and wellbeing and that of their children and families [14, 19]. Misconceptions about contraceptive methods, particularly as related to risk of infertility, discourage young women from using them to delay early pregnancy [14, 20]. Owing to this mix of factors, the rate of adolescent pregnancy has barely declined in the past two decades; in 1993, adolescents comprised 33% of all births or pregnancies compared to 30.8% in 2014 [21].

Despite the seemingly bleak prospects in adolescent sexual and reproductive health, Bangladesh has experienced recent gains in economic development and advancement of women’s rights. In the period from 1991 to 1992 to 2010, the country reduced its poverty rate by nearly half (57% vs 32%) [22]. Progress in girls’ education has resulted in near gender parity for primary school enrollment [23]. And maternal mortality has declined from 569 deaths per 100,000 births in 1990, to 196 deaths per 100,000 live births in 2015 [24]. The disparity between advances in general health and economic factors and stagnant adolescent pregnancy rates suggests a particularly complex set of factors preventing improvement in young girls’ lives.

CARE Bangladesh, with support from the Bill and Melinda Gates Foundation, aimed to design an intervention to address the persistent issue of early birth among young married adolescents in rural Bangladesh. In order to understand the social norms, individual and structural barriers and facilitators to married girls delaying first birth, CARE conducted a comprehensive formative evaluation. Furthermore, we aim to investigate the barriers that married adolescent girls face when opting to pursue alternatives opportunities, such as education or employment, in lieu of immediate childbirth.

## Methods

### Study design and setting

In July and August of 2017, International Centre for Diarrhoeal Disease Research, Bangladesh (icddr,b) researchers conducted in-depth interviews and key informant interviews with community members in two sub-districts: Kurigram Sadar and Rajarhat in Kurigram district in the northern region of Bangladesh. These sites were selected in consultation with the CARE Bangladesh team and local stakeholders on the basis of both accessibility and suitability for intervention. The CARE team also targeted areas of Bangladesh where other partners were not already implementing programs aimed at delaying childbirth among adolescent girls (although other sexual and reproductive health or adolescent programs may exist).

### Study subjects and selection

Based on an extensive desk review on fertility decision-making among adolescents in Bangladesh, researchers wanted to include diverse groups of participants that represented the spheres of influence in the reproductive decision-making of adolescent girls (both married and unmarried girls). These included girls themselves, husbands of married girls, influential adults in the girls’ lives who may advise of control them on fertility decisions, community or religious leaders that may influence the community context for fertility decision-making and medical personnel who may influence girls’ access to appropriate reproductive health care. We estimated that approximately 12 of each sub-group would be a minimum amount, but recruited participants until we reached saturation. We started by recruiting the girls and included as diverse a group a possible for recruitment (in terms of education, age, etc). Girls would then refer their husbands and influential adults, and in both villages, we interviewed all present and available community leaders and medical personnel. It should be noted that the villages from which participant were recruited were fairly homogenous and sub-group diversity was limited.

We recruited adolescent girls under the age of 20 years who were either unmarried (*n* = 20) or newly married within the last 12 months (*n* = 21) as well as husbands of adolescent girls (*n* = 14), “influential adults”^1^ in the girls’ lives, as identified by the girls themselves (*n* = 47), community leaders (*n* = 15) and community level health providers (*n* = 10)^2^ in two villages in Bangladesh. Both married and unmarried girls were included to understand the continuum of support needed for young adolescents as they transition from their natal homes into life as married women and how barriers change at different life stages. Interviews were conducted until data saturation was reached in each category of respondent with the exception of community leaders and health providers; in these categories, all available and willing participants were interviewed.

Within each district, community members were alerted to the study through an announcement by village leaders in the town square. Local data collectors, trained by icddr,b and CARE staff, also asked community leaders to assist them in identifying houses were married and unmarried adolescents lived so that data collection team could individually approach and recruit them to the study. We also identified any other adolescent girls, as well as husbands of adolescents and their family members using snowball techniques whereby community leaders or other adolescent interviewees suggested other potential participants. All available community leaders and health care providers in the area were approached for participation in the study.

Written or oral (depending on literacy) informed consent was collected from all participants prior to their interview. This study was reviewed and received ethical approval by the.

International Review Board of International Centre for Diarrhoeal Disease Research, Bangladesh (icddr,b). For adolescents under the age of 18, we obtained informed consent from a parent/guardian as well as informed assent from the adolescent.

### Data collection and storage

Local interviewers were selected to minimize bias and increase comfort among participants, and interviews were held in Bengali. Female and male participants were also matched to interviewers of the corresponding gender. All interviews were held with just the individual participant in a private area within the village and audio-recorded with the consent of participants. Interviews were conducted using a semi-structured interview guide that was designed based on a literature review, our theory of change, and were pre-tested in the same communities where data collection took place. Interview guides were customized to each sub-group, for a total six unique instruments. At the end of each data collection day, recordings and notes were collated by the research team leader and transported to the icddr,b office. Only relevant members of the research team and program staff had access to these materials throughout the course of the study. No individual names or identifiers of participants were recorded.

### Data analysis

Data from the interviews were transcribed directly in the original language and analyzed for content by a bilingual staff of analysts. Analysis involved coding the data, developing a list of emerging themes, categorizing the themes within a hierarchical framework of main and sub-themes, using a deductive approach. A sample of interviews were double-coded for inter-rater reliability and quality assurance purposes. Where there were discrepancies or disagreements with coding or interpretation, the data analysis team reflected on the text together to determine meaning. All interviews were coded and organized using Dedoose software.

## Results

We conducted a total of 127 in-depth interviews (Table 1). The majority of adolescent girls in this study had completed at least their primary education and had a mean age of 17 years, with a range from 14 to 18 years of age. Income-generating activities were low among this group, particularly among married adolescent girls. Influential adults included mothers, fathers, in-laws and grandparents. Community leaders consisted mainly of village elders. Table 1 further describes the demographic characteristics of each study participant type.

**Table 1:** Basic socio-demographic characteristics of study participants

Participant type	Average age (years)	% completed primary education (class 5)	% involved with income generation	% by religion	% Access to Mobile, Radio, TV
Unmarried adolescent girls (*n* = 20)	17.35	100%	35%	Muslim 95%	Mobile =55% Radio = 15% TV = 40%
Married adolescent girls (*n* = 21)	17.38	95.2%	4.7%	Muslim 100%	Mobile = 57% Radio = 14% TV = 38%
Husbands (*n* = 14)	25.21	78.6%	93%	Muslim 100%	
Influential females (*n* = 25)	39.48	48%	24%	Muslim 96%	
Influential males (*n* = 22)	51.66	45.4%	95.4%	Muslim 100%	
Community leaders (*n* = 15)	49	100.0%	80.0%	Muslim 80%	
Community Health Workers (*n* = 10)	40.2	**% work with govt. FP services department**	**% work with govt. health services department**	**% work with NGO primary healthcare service**	**% with training on adolescent health**
		30% (3)	40% (4)	30% (3)	40% (4)

### Factors that promote early child birth

When examining the factors that promote early childbirth among adolescent girls in this context, several key themes emerged including: stigma of infertility if birth does not occur immediately; presumptions that the married girl is or will be unfaithful to her husband if she does not immediately get pregnant; improved status and family position for girls after giving birth; and social benefits to males once their adolescent wives give birth.

### Stigma of infertility

Among a majority of adolescent girls (both married and unmarried), the desire for early childbearing is strongly fueled by girls’ perceived stigma around delaying child birth. One of the main issues raised by the adolescent girls in this study is the fear that delaying pregnancy and birth will result in them being seen as infertile by the husband, his family or the larger community. Perceived infertility may lead to rejection and sanction of the girl herself and may also jeopardize the standing of her marital family in the eyes of other community members. Girls feel a duty to bear children for the benefit of the husband, who himself may be seen as infertile or impotent if childbearing is delayed.

“*[People would say] there must be some problem, infertile or impotent, that’s why it’s been two years of their marriage but still there is no news of a newborn child”- unmarried adolescent girl, age 17*

*“I will be stigmatized as mistrustful to my husband, my in-laws will not also accept me as their family member. My husband will also be called as impotent and this family will be identified as “bad” family within the community.” - unmarried adolescent girl, age 17*

### Presumptions of infidelity

Efforts to delay birth may also trigger suspicions that the married adolescent girl is having an extra-marital relationship, thus threatening her position within her marital family and leaving her open to potential abuse or derision by her in-laws. Almost all girls in this study preferred to delay child birth, but many reported fearing the rumors of infidelity which would accompany such a choice and the repercussions they may face from the community or family. This leaves girls anxious to prove their fertility and establish their position in their new families with the birth of their first child, despite their desire to delay birth.

*“If any married girl wants to delay her first childbirth, then her husband, mother-in-law, and father-in-law suspect that she had a relationship with someone before she got married and that she is continuing that relationship.” - unmarried adolescent girl, age 17*

*“Many girls don’t want to have children immediately. Husbands think that they might run away with other boys, but if they have children, they cannot leave. To avoid these kinds of suspicious thoughts, girls are having children early.” – married adolescent girl, age 18*

### Girls’ improved status after birth

At the same time that girls feel a stigma *against* delaying birth, several participants (namely males) reported interpersonal benefits for the adolescent girl once she has given birth. According to a number of both husbands of the adolescent girls and other influential males in the community, early birth improves a married girl’s position in her husband’s family and increases her role as a participant in family decisions. Giving birth also endears the married adolescent to her in-laws, particularly the mother-in-law, with whom the couple often lives. Until a married girl has proven her worth through childbirth, she may not be fully embraced as an active member of her marital family.

*“After having a child, the in-laws and the husbands are more caring towards the daughters-in-law. The daughters-in-law have a stronger and a more stable position in the family after the baby is born. They are then able to express their opinions over certain matters.” - influential male, age 57*

*“Now her status has been changed from a wife to a mother. My mother helps her in any work, helps her make her decisions, and, together, they do household chores.” - husband, age 25*

### Social benefits to males after child birth

Husbands themselves also reap a number of personal benefits from having a child soon after marriage, which may not extend to the girl herself. Several male participants mentioned that upon childbirth, a man’s status is elevated, and he is accorded greater respect from his family members as a responsible earner and family man. In some cases, husbands with children are also considered more important within the community at large, being more readily welcomed to participate in social events and decision-making at the community level. Knowledge of these benefits may, in turn, prompt increased pressure from the husband on the adolescent girl to give birth soon after marriage.

*“After becoming a father, people in the area respect a man even more. The community expects more from him. During important community events, he is called on to attend. His presence during various social events is needed.” - influential male, age 60*

### Factors that discourage early child birth

Although girls face a number of real and urgent pressures to give birth immediately upon getting married, many study participants were able to name circumstances which would discourage early birth. Chief among these was a knowledge of the health risks associated with early childbirth and its associated costs, economic benefits of delaying childbirth and opportunities for the couple to develop their marital bond in the absence of children.

### Health risks and associated costs of early childbirth

Fear of complications during pregnancy and childbirth may motivate members of these communities to delay early birth. Despite expressing a strong desire for children right after marriage, a number of both male and female participants acknowledged that early childbearing carries substantial health risks, a fact they had experienced personally or witnessed indirectly. Many participants, including the girls themselves, were able to articulate that pregnancy at a young age increases girls’ risk of a difficult pregnancy or delivery, thereby increasing the likelihood of morbidity and mortality for both the adolescent and her child.

*“I conceived at 17 years, just after 11 months of my marriage. After my childbirth, I found that I was anemic, and I took medicine. During my delivery, I faced severe complications, and I had to be admitted to the hospital.” - married adolescent girl, age 17*

*“One will be out of danger both for mother & child related with delivery complications [if they delay child birth]” - unmarried adolescent girl, age 17*

*“There are lots of benefits to delaying first pregnancy. The mother and the baby will remain healthy. - married adolescent girl, age 18*

In addition to protecting the health of the mother and the child, several participants mentioned that delaying birth could also ease the burden of medical costs associated with complications and risks of child birth at a young age. Early childbirth may require costly emergency obstetric services or follow-up care for newborns and postpartum girls. The majority of participants were able to name theoretical benefits of delaying birth, yet in practice, girls, their husbands and their families may feel intense pressure at individual and community levels to bear children, leaving many to perpetuate the cycle of early birth.

*“One will be enough nourished to get a baby in her womb and if caesarean section is needed then can save 10000-20000BDT to bear the expenses” - married adolescent girl, age 17*

*“Although my wife’s position will be stronger after childbirth at an early age, I will be poor because the baby will be frequently sick and I have to pay for the treatment.” - husband, age 20*

*“Although the society may support delay in pregnancy, in the practical scenario families do not represent this support” – community leader, age 54*

### Economic benefits to delaying childbirth

Many participants, husbands in particular, cited potential economic benefits to delaying child birth that go beyond simply avoiding expenses associated with a high-risk birth. Instead of having a child right away, the adolescent girl could work and generate additional income, strengthening the economic foundation of the family. Postponing birth could also improve long-term financial prospects by allowing time for the family to save money or establish viable income-generating activities. Several participants also mentioned the benefit of delaying the expenses associated with child birth and rearing, which could further contribute to the long-term financial stability of the family.

*While delaying, she could become engaged in some kind of an income generating activity. She can also use this time to organize and strengthen her family.” - married adolescent girl, age 18*

*“Due to my wife delaying first childbirth, I was able to save money for a few years and establish a grocery shop in my village. Through this shop, I can generate regular income. After establishing my grocery shop, I could better manage all the costs related to the pregnancy of my wife, such as medicine and ultrasound tests.” - husband, age 25*

*“I am under pressure after the childbirth. Before marriage it was only me, after marriage we are two, and after childbirth we are three. Now I have to increase my income from two taka to three taka” - husband, age 25*

### Opportunities for marital and maternal readiness

In addition to the economic and health benefits, about half of the participants felt that delaying birth could have important interpersonal benefits for the girl and her husband by allowing them more time to build their marital bond. This time spent as a couple, without children, is seen as integral to strengthening the foundation of the marriage and improving long-term compatibility. Moreover, several of the young girls themselves expressed feeling ill-equipped to provide care to a baby so early in life, as they themselves are only just children. These girls conveyed wanting more time to mature both mentally and physically in preparation for becoming a mother.

*“If one can delay pregnancy then one will get enough time to get to know the likes and dislikes of the husband and vice versa” - married adolescent girl, age 17*

*As I am a child, how will I be able to care for my own child? I don’t even know how to take care of a child.” - married adolescent girl, age 16*

*“Delaying child birth will provide enough time to learn proper child care, also my body will be matured enough to carry the child for 9 months” - married adolescent girl, age 17*

#### Girls’ economic and educational development as a strategy to delay birth

Participants were asked to describe their support for girls’ participation in education and income-generating activities. Nearly half of participants approved of these types of opportunities and viewed them as having tangible benefits to the adolescent and her family. Adolescent girls, in particular, felt that pursuit of education or labor participation could be a strategy to convince key individuals in their lives (namely the mother-in-law) to allow them to delay birth for a period of time following the marriage. While not the majority, a few participants also thought that increased earnings potential or status associated with higher education could help counter community stigma associated with delaying early birth among young girls.

*“[If I am earning income] I will not be forced to get pregnant. My mother-in-law can be an obstacle to me, but if I can make her understand that my involvement in work will bring money, she will not create a problem. If the training is near my house, it would be advantageous to me.” - married adolescent girl, age 19*

*“In our community, education is valued highly. Our community respects educated people.” married adolescent girl, age 18*

*“When people will see that I am earning money also and helping in laws then after a certain time community people will be quiet, they will not say bad things to us as they used to do for the reason of our delayed pregnancy” unmarried adolescent girl, age 17*

### Challenges to girls’ pursuit of educational and economic opportunities

Several obstacles complicate married adolescent girls’ pursuit of educational and economic alternatives to early childbirth, including severe restrictions to girls’ mobility and fear that the girl will become “spoiled” through exposure to the outside world. Girls also lack agency for decision-making, living in a world where limits and freedoms are dictated by the husband or the marital family (in particular the mother-in-law).

#### Lack of girls’ mobility

Many participants in this study noted that married adolescents’ movements are severely restricted once they are in the marital home. Girls are forbidden from traveling outside of the home on their own, even to visit their parents or relatives. Furthermore, any travel must be approved by either the husband or the mother-in-law. A girl’s mobility becomes even more restricted after she has a child, as she is expected to stay home to care for the baby, everyone else in the household, and also complete her own chores.

In many cases, there is an underlying fear that the girl may become “spoiled” by exposure to the outside community and participation in new experiences. Community norms and the specter of collective judgment often leave married adolescent girls confined to the marital household for a prescribed period of time following the union. A number of participants noted a strong paranoia on the part of the marital family that the married adolescent would be corrupted by any time spent away from the home, which would reflect poorly upon and affect the status of the marital family. These beliefs are often reinforced by the public policing of girls’ activities by the community at large. Even in the cases that married girls are granted permission to go outside the home, activities are usually restricted to the immediate surroundings.

*“I do not go anywhere, even places near my house, because my mother-in-law suspects that if I talk to anybody, I might be badly influenced by them.” - married adolescent girl, age 18*

*“A newly married wife cannot go outside of the home before being married for at least six months. Everybody sees it negatively if a newly married wife goes out to work. In-laws do not allow that either. After a while, they might send the daughter-in-law outside for some small jobs, like taking care of the cattle.” - husband, age 23*

*“There are some families that do not allow their daughters-in-law to work outside home. The daughters-in-law should not go outside, let them stay inside the house, because, if she goes out, she will become spoiled. If she goes out, she will become too clever. This is because, there are some wives who go outside, learn many new things, and then humiliate the in-laws.” - married adolescent girl, age 18*

*“I will not allow my daughter-in-law to go outside of the locality. Community people will say bad things about my newly married daughter-in-law and about the family, so I can’t let that happen. If she wants to work somewhere within the village, then that will be alright.” - influential female, age 45*

#### Lack of girls’ individual agency

A number of both married adolescents and husands in this study mentioned that in order to participate in activities outside of the home, married adolescents must often receive approval from their husbands and marital families. This lack of agency was the norm across both of the communities in this study and was seen as a means of keeping cohesion and strength within the family unit. The mother-in-law plays an especially strong role in upholding traditional norms and policing married adolescents’ behavior. In many cases, the mother-in-law is the final decision-maker when it comes to adolescent girls’ pursuit of education or income-generating activities, in some instances overriding the wishes of the married adolescent’s own parents. Even in cases where girls are permitted to work, the type and location of the labor is often dictated by the husband or others in the marital family. These realities may undercut any strategy of using education or earnings as an inducement to loosening the marital family’s grip of control over a married adolescents’ life.

*“My mother-in-law does not want me to continue my education. My mother-in-law says that there is no need for education. I cannot go to school regularly; I still want to finish my studies. My father says that he will give me money to finish my studies, but my mother-in-law will not allow me to continue my studies. She says there is no need for me to study anymore. ‘Whatever you have studied so far is enough,’ she says.” - married adolescent girl, age 16*

*“I wanted to be a nurse, but it would not happen. I have dropped out of school. My mother-in-law and husband will not allow me to restart my education. In my family, my mother-in-law is the primary decision maker. Even for training, she and my husband will not allow me to go to a distant place.” - married adolescent girl, age 14*

*“Since I am the one that allows her to do income activities at the home, my family members will not speak ill to her” - husband, age 25*

### Barriers to contraceptive use among adolescent girls

Several factors complicate adolescent girls’ access to and use of contraceptive use. Girls’ low degree of mobility and lack of decision-making power limit their capacity to engage with health services and make free and informed choices about contraceptives use. Furthermore, the persistence of misinformation and misconceptions around contraceptive methods may discourage use among women in these communities.

### Low mobility and decision-making power for health

As with access to education and income-generating activities, lack of mobility and low decision-making power also restrict married adolescent girls’ access to health centers where they may receive contraceptive services. Newly married adolescent girls who are seen “moving around” on their own may be subject to judgment in the community context. Moreover, married adolescents reported that the husbands decide if and when to use a family planning method. After some time in the marital union, however, it may become easier for a married adolescent girl to negotiate some measure of independence and mobility to access health care.

*“People would say that the new daughter-in-law is going alone to collect contraceptive pills. Village people would say that the new daughter-in-law already started moving to and fro.”- married adolescent girl, age 16*

*“Use of a family planning method solely depends on my husband’s decision; in this case, my decision is not counted.” - married adolescent girl, age 14*

*“I am the new daughter-in-law; I will not be allowed to go outside alone. The girls who are married for a while can go alone to the health center; their husbands will not stop them.” - married adolescent girl, age 14*

### Misconceptions around contraception

Strong misconceptions about the possible side effects of modern methods also discourage married adolescent girls from using contraception to prevent a first pregnancy. A number of female participants in this study, including adolescent girls as well as influential female adults, displayed a lack of proper knowledge and understanding of contraceptive methods, their applications and their side effects. Foremost among these misconceptions is the belief that the use of any contraception can lead to infertility. Participants also feared injury or mortality due to use of modern contraceptive methods. These misconceptions are reinforced by influential people in the girls’ lives, as they are discouraged or even forbidden to use contraceptives by parents, husbands and friends.

*“The flower (placenta) will dry out if one take contraceptive before first birth” - married adolescent girl, age 17*

*“After marriage I heard that condoms were not good. In the past, I did not understand anything about this. In many instances, condoms can be inserted into the body, and, if inserted, a person can die.” - married adolescent girl, age 18*

*“Mothers-in-law, mothers, and elderly people in the village forbid taking oral contraceptive pills before the birth of one’s first child, because, if pills are taken, the uterus will die, and, consequently, the woman may not conceive. They advise us to take pills or injections or whatever we like after our first child.” - influential female, age 35*

## Discussion

This study provides an understanding of context-specific drivers of early birth among married adolescent girls in Bangladesh, particularly from the individual, family and community spheres. It revealed a host of factors that inhibit newly married adolescent girls from delaying childbirth and pursuing educational and economic alternatives. Married adolescent girls often have children soon after marriage to prove their fertility, please their in-laws, and establish their position in the family and community. Fear of stigma around infidelity, a lack of access to and appropriate knowledge of contraceptive methods and severe limitations in mobility and decision-making power further decrease girls’ ability to prevent childbirth soon after marriage.

While education and income-generating activities for adolescent girls are valued in this community, their importance does not necessarily supersede the marital obligations of childrearing and domestic chores. Girls themselves placed a great emphasis on continuing their education and working to generate income. Husbands also praised the potential economic benefits of a young wife’s financial contributions to the family. However, traditional norms may limit girls’ ability to delay childbearing once they are married.

The mother-in-law in Bangladesh plays a particularly strong role as household decision-maker and holds considerable power over the day-to-day life of the married adolescent. Study participants cited the mother-in-law as a main source of pressure on the married adolescent to produce an offspring immediately following marriage, and a major barrier to using contraceptive methods. She controls the married girl’s day-to-day activities, dictating chores and monitoring her access to the outside world. In some cases, girls may even suffer verbal abuse at the hands of an aggrieved in-law. The importance of the mother-in-law’s influence in relation to married adolescent’s timing of birth, use of health services (namely, family planning) and participation in activities outside of the marital home has been well-documented in Bangladesh and other similar contexts [12–16]. As such, effective programming strategies should consider the participation, or at the very least acknowledgement, of the mother-in-law in designing interventions to delay early birth.

An underlying current of gender inequality and a lack of value for girls’ lives places the problem of early childbearing among married adolescent girls in the broader context of women’s empowerment issues [25]. Despite knowledge of the risks associated with adolescent pregnancy and childbirth among these communities, the majority of respondents in a married girl’s life uphold traditions that dictate girls become pregnant immediately following childbirth. These key players, namely the husband or in-laws, severely limit her mobility and capacity to make independent decisions about her health and life options. Community norms further reinforce the suppression of girls’ autonomy and equality by perpetuating rumors around infertility and infidelity for those who choose to delay birth. Addressing gender-based bias and lack of individual agency for adolescent girls is critical to the success of any reproductive health, economic or educational intervention in this context [26, 27].

These findings should be viewed in light of the study limitations. Given the purposive nature of participant selection, the findings are not generalizable. The contextual barriers identified here are specific to the Kurigram region and may not apply to other parts of Bangladesh. Finally, due to the highly sensitive topic of sexual and reproductive health in this context, there may be the presence of response bias among some participants.

## Conclusion

The problem of early childbearing in Bangladesh has roots in a number of socio-cultural and structural norms. A multi-faceted intervention that addresses this constellation of issues has the best chance of increasing girls’ ability to delay childbirth and providing them with viable alternative educational and economic pursuits.

Based on the study findings, there are a number of structural, social and individual changes that must be made to promote the delay of early childbirth among newly married adolescent girls. Health systems must be strengthened to provide more accessible and youth-friendly reproductive health services to nulliparous married adolescents. Community level education and behavior change activities are needed to combat negative social norms and misconceptions about different methods of family planning and delaying early pregnancy. Key stakeholders, such as husbands, in-laws and influential community leaders should also be involved in creating an enabling environment for interventions aimed at improving young girls’ lives. Girls themselves can be supported through programs that increase gender parity and their individual agency to use contraceptives and to participate in educational and income-generating activities [26].

## Data Availability

The datasets used and/or analyzed during the current study are available from the corresponding author on reasonable request.

## Abbreviations

icddr,b: International Centre for Diarrhoeal Disease Research, Bangladesh

## Footnotes

1 “Influential adults” are those to whom adolescent girls may turn for advice or guidance on important life decisions such as marriage and birth. To identify influential adults, after each adolescent girl was interviewed, we her to name two adults in her life that she considered influential (based on the described criteria). We then sought out these individuals for independent interviews. In most cases we interviewed at least one influential adult per girl.

2 Although we attempted to recruit a minimum number of 12 participants for each subgroup, we spoke with all available health providers in the region during the period of data collection and were able to reach *n* = 10.

## References

[1] UNFPA (2015). Girlhood, not motherhood: preventing adolescent pregnancy.

[2] Godha D, Hotchkiss DR, Gage AJ (2013). Association between child marriage and reproductive health outcomes and service utilization: a multi-country study from South Asia. J Adolesc Health.

[3] Ganchimeg T, Ota E, Morisaki N, Laopaiboon M, Lumbiganon P, Zhang J (2014). Pregnancy and childbirth outcomes among adolescent mothers: a W orld health organization multicountry study. BJOG Int J Obstet Gynaecol.

[4] Karim N, Greene M, Picard M (2016). The cultural context of child marriage in Nepal and Bangladesh: Findings from CARE’s Tipping Point Project. Community participatory analysis. Research report.

[5] Raj A (2010). When the mother is a child: the impact of child marriage on the health and human rights of girls. Arch Dis Child.

[6] Henry EG, Lehnertz NB, Alam A, Ali NA, Williams EK, Rahman SM, Ahmed S, El Arifeen S, Baqui AH, Winch PJ (2014). Sociocultural factors perpetuating the practices of early marriage and childbirth in Sylhet District, Bangladesh. Int Health.

[7] Khan R, Kerry LD, MacQuarrie QN, Sultana M (2016). The Men Are Away: Pregnancy Risk and Family Planning Needs among Women with a Migrant Husband inBarisal, Bangladesh. DHS Further Analysis Reports No. 98.

[8] Human Rights Watch (2015). Marry before your house is swept away: Child marriage in Bangladesh.

[9] Lemmon GT, El Harake LS (2014). Child Brides, Global Consequences: How to End Child Marriage. Council on Foreign Relations.

[10] Char A, Saavala M, Kulmala T (2010). Influence of mothers-in-law on young couples’ family planning decisions in rural India. Reprod Health Matters.

[11] Upadhyay P, Liabsuetrakul T, Shrestha AB, Pradhan N (2014). Influence of family members on utilization of maternal health care services among teen and adult pregnant women in Kathmandu, Nepal: a cross sectional study. Reprod Health.

[12] Ali TS, Krantz G, Gul R, Asad N, Johansson E, Mogren I (2011). Gender roles and their influence on life prospects for women in urban Karachi, Pakistan: a qualitative study. Glob Health Action.

[13] Shahabuddin AS, Nöstlinger C, Delvaux T, Sarker M, Bardají A, De Brouwere V, Broerse JE (2016). What influences adolescent girls’ decision-making regarding contraceptive methods use and childbearing? A qualitative exploratory study in Rangpur District, Bangladesh. PloS one.

[14] Gipson JD, Hindin MJ (2007). ‘Marriage means having children and forming your family, so what is the need of discussion?‘communication and negotiation of childbearing preferences among Bangladeshi couples. Cult Health Sex.

[15] Kumar A, Bordone V, Muttarak R (2016). Like mother (−in-law) like daughter? Influence of the older generation’s fertility behaviours on women’s desired family size in Bihar, India. Eur J Popul.

[16] Haider S, Todd C, Ahmadzai M, Rahimi S, Azfar P, Morris J, Miller S (2009). Childbearing and contraceptive decision making among afghan men and women: a qualitative analysis. Health Care Women Int.

[17] Balk D (1997). Defying gender norms in rural Bangladesh: a social demographic analysis. Popul Stud.

[18] Schuler SR (2007). Rural Bangladesh: Sound policies, evolving gender norms, and family strategies. Exclusion, Gender and Education: Case Studies from the Developing World.

[19] Rashid SF (2006). Small powers, little choice: contextualising reproductive and sexual rights in slums in Bangladesh. IDS Bull.

[20] Uddin MJ, Choudhury AM (2008). Reproductive health awareness among adolescent girls in rural Bangladesh. Asia Pac J Public Health.

[21] Islam MM, Islam MK, Hasan MS, Hossain MB (2017). Adolescent motherhood in Bangladesh: trends and determinants. PLoS One.

[22] Bangladesh Planning Commission (2013). Millennium development goals: Bangladesh progress report-2015. General Economics Division. Government of People’s Republic of Bangladesh, Dhaka.

[23] UNICEF (2015). State of the world’s children 2015: executive summary.

[24] Bongaarts J (2016). WHO, UNICEF, UNFPA, World Bank Group, and United Nations Population Division Trends in Maternal Mortality: 1990 to 2015 Geneva: World Health Organization, 2015. Population Dev Rev.

[25] Schuler SR, Rottach E (2010). Women’s empowerment across generations in Bangladesh. J Dev Stud.

[26] Barker G, Ricardo C, Nascimento M, Olukoya A, Santos C (2010). Questioning gender norms with men to improve health outcomes: evidence of impact. Global Public Health.

[27] Kraft JM, Wilkins KG, Morales GJ, Widyono M, Middlestadt SE (2014). An evidence review of gender-integrated interventions in reproductive and maternal-child health. J Health Commun.

